# Abnormalities of Brain White Matter in Type 2 Diabetes Mellitus: A Meta-Analysis of Diffusion Tensor Imaging

**DOI:** 10.3389/fnagi.2021.693890

**Published:** 2021-08-06

**Authors:** Li Huang, Qingqing Zhang, Tong Tang, Minguang Yang, Cong Chen, Jing Tao, Shengxiang Liang

**Affiliations:** ^1^National-Local Joint Engineering Research Center of Rehabilitation Medicine Technology, Fujian University of Traditional Chinese Medicine, Fuzhou, China; ^2^Rehabilitation Industry Institute, Fujian University of Traditional Chinese Medicine, Fuzhou, China; ^3^College of Rehabilitation Medicine, Fujian University of Traditional Chinese Medicine, Fuzhou, China; ^4^Traditional Chinese Medicine Rehabilitation Research Center of State Administration of Traditional Chinese Medicine, Fujian University of Traditional Chinese Medicine, Fuzhou, China

**Keywords:** type 2 diabetes mellitus, diffusion tensor imaging, white matter, meta-analysis, activation likelihood estimation

## Abstract

**Aims:** The study aimed to conduct a meta-analysis to determine the abnormalities of white matter in patients with type 2 diabetes mellitus (T2DM) by identifying the consistency of diffusion tensor imaging (DTI).

**Method:** The literature for DTI comparing patients with T2DM with controls published before October 30, 2020, were reviewed in PubMed, Web of Science, Embase, CNKI, and Wan Fang databases. The meta-analysis was performed using the activation likelihood estimation (ALE) method, including 12 reports and 381 patients with T2DM.

**Results:** The meta-analysis identified 10 white matter regions that showed a consistent reduction of fractional anisotropy (FA) in patients with T2DM, including genu of the corpus callosum, the body of corpus callosum, bilateral anterior corona radiata, bilateral superior corona radiata, bilateral cingulum, and bilateral superior fronto-occipital fasciculus.

**Conclusion:** This study revealed the abnormal characteristics of white matter in T2DM, which would be helpful to understand the underlying neuropathological and physiological mechanisms of T2DM and provide evidence for clinical diagnosis and treatment.

## Introduction

Type 2 diabetes mellitus (T2DM) is a chronic metabolic disease in which blood glucose is abnormal due to insufficient insulin secretion or the inability of the body to effectively use insulin (Arnold et al., 2018). According to the International Diabetes Federation (IDF), the incidence of type 2 diabetes worldwide is estimated to be 8.18%, with 415 million adults living with the disease and the number still on the rise (Zheng et al., 2018). T2DM is recognized as an independent risk factor for cognitive impairment (Biessels et al., 2006; Cukierman-Yaffe et al., 2020). The risk of developing Alzheimer's disease (AD) in patients with T2DM is two times as high as that in healthy individuals (Peila et al., 2002; Crane et al., 2013). Studies have shown that cognitive decline is related to abnormal brain microstructures (Alfaro et al., 2018; Reas et al., 2020). Clarifying these brain abnormalities is helpful for the clinical diagnosis and treatment of cognitive decline in T2DM.

White matter makes up about 50% of the volume in the human brain (Filley, 1998). In the human forebrain, there are about 135,000 km of myelinated fibers (Saver, 2006) connecting the gray matter regions. Information processing in gray matter, manifested by synaptic events such as neurotransmitter release and long-term enhancement, is supplemented by information transmission in white matter, both of which are essential for the highly evolved behavior of the human brain (Filley, 2010). However, several studies have found abnormalities in the white matter of T2DM (Gao et al., 2019; Zhuo et al., 2019; Cui et al., 2020).

As a highly sensitive method for detecting the microstructure of white matter, diffusion tensor imaging (DTI) is an important technique for assessing white matter integrity *in vivo* (Catani, 2006; Mori et al., 2009). Based on the diffusion velocity of water molecules in different tissues, white matter integrity is usually estimated *via* fractional anisotropy (FA), and diffusion coefficient parameters are calculated by DTI. Previous DTI studies have observed widespread white matter abnormalities in patients with type 2 diabetes, but the results of these studies were mixed. For example, a reduction in FA was found mainly in the left temporal lobe (Yau et al., 2009), as opposed to a reduction in the right temporal lobe (Xiong et al., 2019b). Other researchers have also found a decrease in FA in the frontal lobes and corpus callosum (Ruan et al., 2017; Xie et al., 2017; Su et al., 2020). Differences in the results of these studies might be due to the limited sample sizes, different acquisition parameters, and analysis methods. Several systematic reviews have summarized the brain microstructural alterations in T2DM using DTI, which suggests the detrimental effects of T2DM on cognitive functions that might be associated with the alterations in brain microstructure (Sanjari Moghaddam et al., 2019; Alotaibi et al., 2021). Although the specific abnormal regions of white matter in T2DM were summarized in the systematic reviews, the locations and sizes of the alterations were not reported. Therefore, it is urgently needed to conduct a meta-analysis to identify the locations of the alterations in white matter in patients with T2DM.

Activation likelihood estimation (ALE) is a brain imaging meta-analysis method based on coordinates, which integrates many brain imaging results to obtain locations of the stable differences in brain regions through automated statistical analysis (Turkeltaub et al., 2002; Laird et al., 2005; Eickhoff et al., 2009, 2012). To date, ALE has been widely used in neuroimaging studies of neurological and psychological disorders (Tahmasian et al., 2017; Xia et al., 2017; Roberts et al., 2020). To identify the consistency of white matter changes in T2DM, we applied ALE to conduct a meta-analysis of DTI in patients with T2DM.

## Materials and Methods

### Search Strategy

A meta-analysis of the literature on alterations in the white matter of T2DM was performed according to PRISMA guidelines (Moher et al., 2015). All the articles published before October 30, 2020, were retrieved from PubMed, Web of Science, Embase, CNKI, and Wan Fang database. Two researchers independently used the combined keywords to complete the search: (1) “White matter,” “Diffusion tensor imaging,” or “fractional anisotropy,” and (2) “Type 2 diabetes mellitus” or “T2DM.” The FA differences were estimated in most studies *via* voxel-based analysis (VBA) or tract-based special statistics (TBSS), while the comparison of other diffusion measures was very limited, such as mean diffusivity (MD), radial diffusivity (RD), and axial diffusivity (AD). We comprehensively searched the studies using MD, RD, or AD for DTI analysis in T2DM. There was one study that estimated MD and one that estimated RD which met the inclusion criteria. Therefore, this study conducted an imaging meta-analysis to investigate the FA differences.

### Study Inclusion and Exclusion Criteria

Studies were included according to the criteria: (1) the original research articles were published in peer-reviewed journals, (2) patients with T2DM were diagnosed, (3) significant differences in FA values were examined in the study, and (4) the coordinates in Montreal Neurological Institute (MNI) or Talairach (Tal) space were reported.

Studies were excluded in the following situations: (1) the study included patients with undiagnosed T2DM in the experimental group, (2) coordinates were not reported in the study or were not obtained after contacting the first author, (3) the results were limited to a particular region of interest, not the whole brain, and (4) the articles or non-research articles were unpublished.

### Literature Quality Assessment

The literature quality assessment form was developed based on the previous study (Iwabuchi et al., 2015). The total score of the form was 20, with two parts of sample information (10 points) and imaging methods (10 points).

Sample information was scored as follows: (1) patients were diagnosed with specified standardized diagnostic criteria (1 point), (2) age and educational background were reported with mean (or median) and SDs (or range) (2 points), (3) healthy comparison subjects were evaluated to exclude psychiatric and medical illnesses, and demographic data were reported (1 point), (4) important clinical variables [e.g., illness duration, fasting plasma glucose (FPG) level, HbA1c, and cognitive level] were reported with mean (or median) and SDs (or range) (4 points), and (5) sample size per group was ≥10 (2 points).

Evaluation of imaging methods was scored in the following aspects: (1) whole-brain analysis was automated without *a priori* regional selection (3 points), (2) magnet strength was at least 1.5 T (1 point), (3) the study had at least six of diffusion direction (1 point), (4) whole-brain coverage of scans was used (1 point), (5) the acquisition and preprocessing techniques were clearly described so that they could be reproduced (1 point), (6) coordinates were reported in standard space (1 point), (7) significant results were reported after correction for multiple testing using a standard statistical procedure [false discovery rate (FDR), family-wise error (FWE), or permutation-based methods] (1 point), and (8) conclusions were consistent with the results obtained and the limitations were discussed (1 point).

### Data Extraction

Information extraction from the included study was done independently by two researchers. We extracted the baseline information, DTI characteristics, and coordinates in the study. Baseline information included the name of the first author, publication year, study group, and sample size of each group, age, years of education, FPG, HbA1c, disease course, body mass index (BMI), and cognitive level. DTI characteristics included the following information: magnet, image processing, analytical method, coordinates type, direction, b values, and statistical threshold.

### Coordinate-Based Meta-Analysis

To analyze the consistency of abnormal FA clusters reported in all studies, a meta-analysis of ALE was performed using the GingerALE2.3.3 software package (Laird et al., 2005; Eickhoff et al., 2009; Research Imaging Institute of the University of Texas Health Science Center, San Antonio, TX, United States). The voxel coordinates of each study were regarded as probability distributions to create ALE distribution maps (Turkeltaub et al., 2002). The x, y, and z peak activation coordinates of all the white matter fiber clusters were included as the input for the meta-analysis. FA measures from all included studies were used in the meta-analysis. The analysis was performed in the MNI space. The ALE meta-analysis was estimated using a cluster-level inference threshold of *p* < 0.05 (FWE correction) with 5,000 permutations and *p* < 0.05. Then, the Johns Hopkins University (JHU) DTI-based white-matter atlases were applied to label the resulting clusters. Besides, Colin brain template in MNI space was used to visualize the results using DPABI software (Yan et al., 2016).

## Results

### Search Results and Study Characteristics

**Figure 1:**
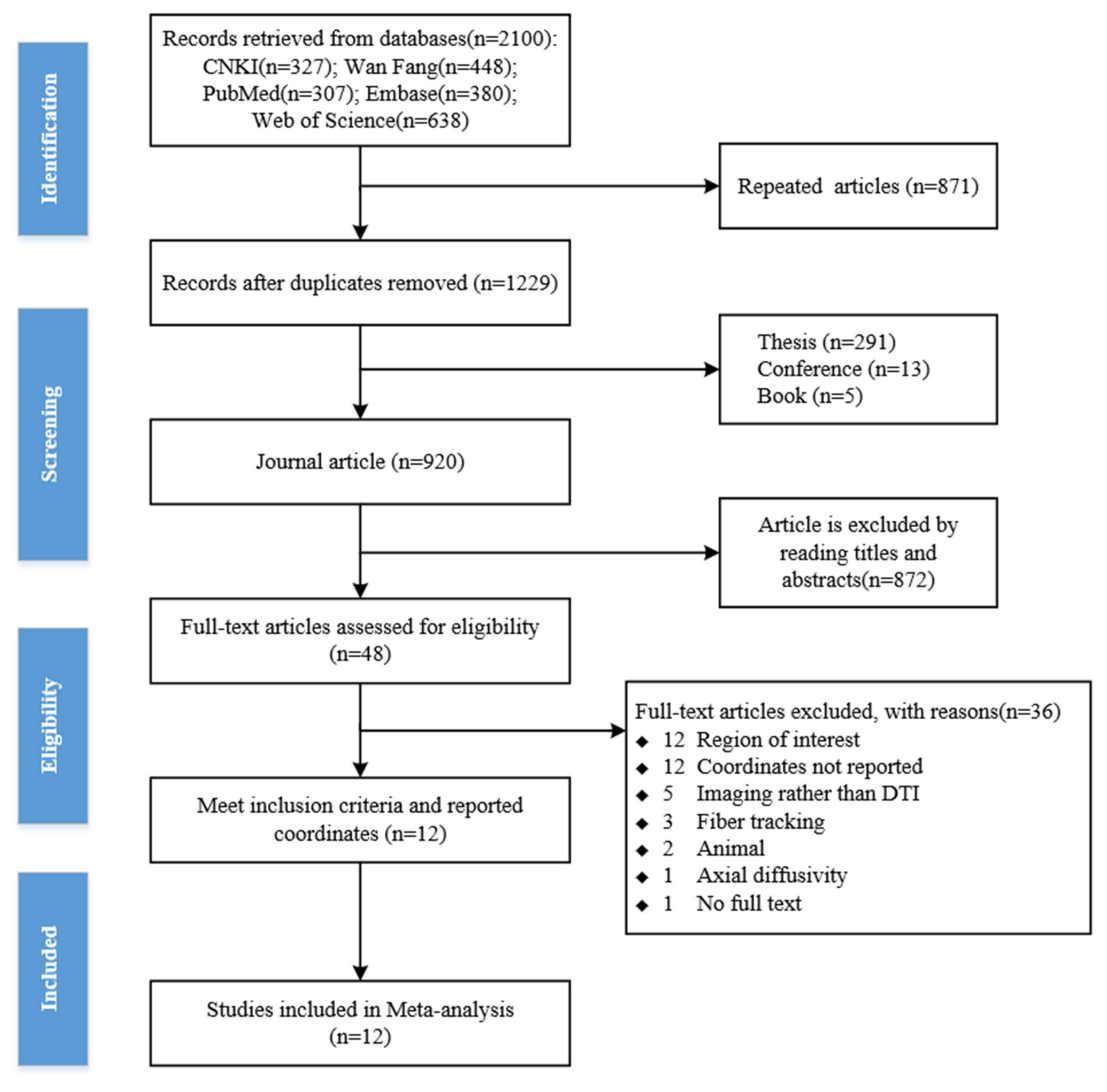
The flowchart of the literature search.

Two researchers independently searched the literature according to the search terms. A total of 2,100 articles were retrieved using the search strategy (CNKI *n* = 327; Web of Science *n* = 638; Wan Fang *n* = 448; PubMed *n* = 307; Embase *n* = 380). About 871 duplicate papers were deleted, 291 theses, 13 conference papers, and 5 books were excluded according to literature types, and 920 journal articles were selected. The titles or abstracts of journal papers were screened according to the inclusion and exclusion criteria, and 48 journal articles were selected to meet the criteria. Two researchers independently reviewed the full text of 48 articles. About 36 articles were excluded for the following reasons: region of interest, coordinates not reported, imaging rather than DTI, fiber tracking, axial diffusion, animal, and full text not found. Finally, a total of 12 studies were included for the meta-analysis (Yau et al., 2009, 2010, 2013; Chen et al., 2013; Kim et al., 2016; Nouwen et al., 2017; Ruan et al., 2017; Xie et al., 2017; Yoon et al., 2017; Liang et al., 2019; Xiong et al., 2019b; Su et al., 2020; Figure 1).

**Figure 2:**
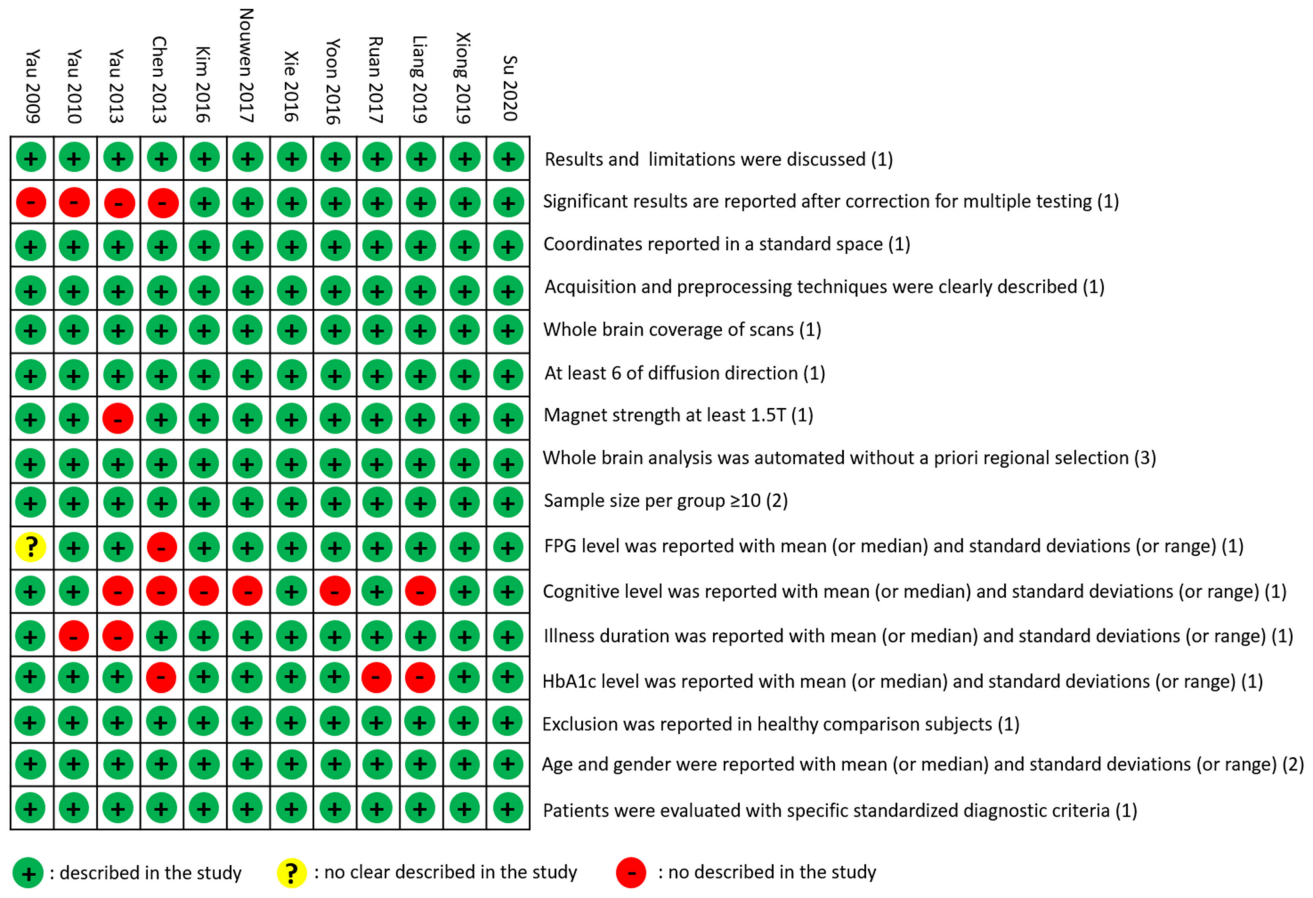
Literature quality assessment. The green circle means the information is clearly described in the study. The yellow circle means the information is not clearly described in the study. The red circle means the information is not described in the study.

The T2DM group and the control group in the 12 included studies were usually described by their characteristics, such as age, gender, education, FPG, HbA1C, duration, BMI, and cognitive level (Table 1). In the included studies, one study (Kim et al., 2016) showed a higher level of HbA1C than the others. The DTI parameters and analysis methods in the studies were used to examine magnetic field intensity, toolbox, analysis method, coordinate space, the number of diffusion direction, b0-value, and statistical threshold (Table 2). Literature quality assessments with a total score of 20 were conducted. All the scores of the 12 studies were higher than 16, which indicated that the quality of the included studies was acceptable (Figure 2).

**Table 1:** Study and subject information of the studies included in the current meta-analysis.

**References**	**Groups**	**Sample**	**Age (years)**	**Education (years)**	**FPG (mmol/L)**	**HbA1C (%)**	**Duration (years)**	**BMI (kg/m^2^)**	**Full Scale IQ**	**MMSE**	**MoCA**
Yau et al. (2009)	T2DM	24	57.21 ± 8.05	15.29 ± 2.76	NA	7.83 ± 1.88	7.94 ± 5.64	32.13 ± 5.96	106.74 ± 13.00	NA	NA
	Control	17	56.44 ± 6.94	16.06 ± 1.75	NA	5.37 ± 0.42	—	24.09 ± 3.69	112.78 ± 12.28	NA	NA
Yau et al. (2010)	T2DM	18	16.46 ± 1.89	10.75 ± 1.53	8.37 ± 4.79	8.32 ± 2.87	NA	37.70 ± 6.36	87.83 ± 12.55	NA	NA
	Control	18	17.16 ± 1.45	11.15 ± 1.66	4.16 ± 0.43	5.29 ± 0.33	—	36.80 ± 7.22	103.63 ± 11.75	NA	NA
Yau et al. (2013)	HTN/T2DM	22	58.36 ± 7.91	NA	8.01 ± 3.26	7.89 ± 1.90	NA	32.89 ± 7.03	NA	NA	NA
	HTN/non-T2DM	11	62.38 ± 7.32	NA	4.40 ± 0.62	5.64 ± 0.19	—	25.48 ± 5.08	NA	NA	NA
Chen et al. (2013)	T2DM	16	61.00 ± 12.00	NA	NA	NA	13.00 ± 4.00	NA	NA	NA	NA
	Control	16	60.00 ± 8.00	NA	NA	NA	—	NA	NA	NA	NA
Kim et al. (2016)	T2DM	20	54.60 ± 2.30	11.90 ± 2.30	10.00 ± 1.04	10.70 ± 0.30	12.10 ± 6.50	24.70 ± 0.60	NA	NA	NA
	Control	20	54.30 ± 2.40	10.00 ± 3.60	5.19 ± 0.13	5.90 ± 0.10	—	23.60 ± 0.40	NA	NA	NA
Nouwen et al. (2017)	T2DM	13	16.00 ± 1.60	NA	8.87 ± 3.87	7.80 ± 1.97	2.57 ± 1.92	NA	NA	NA	NA
	Control	20	16.10 ± 1.90	NA	4.78 ± 0.49	5.29 ± 0.33	—	NA	NA	NA	NA
Xie et al. (2017)	T2DM	58	56.09 ± 8.16	11.72 ± 3.31	8.06 ± 2.81	8.35 ± 2.10	7.60 ± 5.82	25.57 ± 2.19	NA	29.21 ± 0.89	NA
	Control	58	54.66 ± 7.03	11.07 ± 2.64	5.13 ± 0.65	5.56 ± 0.33	—	24.64 ± 2.99	NA	29.50 ± 0.94	NA
Yoon et al. (2017)	T2DM	100	49.20 ± 7.70	NA	7.67 ± 2.24	7.12 ± 1.43	1.83 ± 1.53	25.50 ± 3.40	NA	NA	NA
	Control	50	49.00 ± 7.80	NA	5.24 ± 0.21	5.29 ± 0.15	—	22.70 ± 1.80	NA	NA	NA
Ruan et al. (2017)	T2DM	30	55.83 ± 6.00	7.10 ± 1.30	12.39 ± 2.44	NA	8.77 ± 2.46	NA	NA	NA	24.77 ± 1.04
	Control	30	56.33 ± 5.27	7.23 ± 1.33	4.88 ± 0.53	NA	—	NA	NA	NA	28.47 ± 1.31
Liang et al. (2019)	T2DM	34	58.29 ± 4.19	10.50 ± 1.21	6.64 ± 2.00	7.85 ± 1.34	6.85(3-14)	24.36 ± 1.89	NA	NA	NA
	Control	32	56.31 ± 4.46	9.75 ± 1.32	5.28 ± 0.23	NA	—	20.58 ± 1.47	NA	NA	NA
Xiong et al. (2019b)	T2DM-MCI	20	63.55 ± 5.81	11.30 ± 3.61	9.39 ± 2.16	8.15 ± 1.63	9.09 ± 8.14	24.37 ± 3.32	NA	25.40 ± 2.09	25.25 ± 1.25
	Control	28	59.65 ± 5.98	10.18 ± 3.07	5.19 ± 0.68	5.23 ± 0.38	—	24.03 ± 2.02	NA	28.36 ± 0.99	28.64 ± 1.22
Su et al. (2020)	T2DM	26	55.58 ± 5.93	14.54 ± 1.61	9.25 ± 3.38	8.13 ± 1.76	11.12 ± 5.56	23.97 ± 2.22	NA	28.69 ± 1.29	27.50 ± 1.24
	Control	25	53.12 ± 4.79	15.56 ± 2.00	5.31 ± 0.85	5.58 ± 0.51	—	24.00 ± 3.45	NA	28.56 ± 1.42	27.54 ± 1.10

*Data are mean ± SD. BMI, body mass index; FPG, fasting plasma glucose; HTN, hypertension; MCI, mild cognitive impairment; MMSE, mini-mental state examination; MoCA, montreal cognitive assessment; T2DM, type 2 diabetes mellitus*.

**Table 2:** Diffusion tensor imaging (DTI) parameters and analysis methods in the studies of the meta-analysis.

**References**	**Magnet**	**Image processing**	**Analytical method**	**Coordinates**	**Direction**	**b0 (s/mm^2^)**	**Statistical threshold**
Yau et al. (2009)	1.5T	MIDAS, ART2	VBA	Tal	6	1,000	*p* < 0.005
Yau et al. (2010)	1.5T	SPM	VBA	Tal	6	1,000	*p* < 0.005
Yau et al. (2013)	NA	ART2	VBA	Tal	6	1,000	*p* < 0.01
Chen et al. (2013)	3.0T	SPM	VBA	MNI	15	1,000	*p* < 0.01
Kim et al. (2016)	3.0T	FSL	TBSS	MNI	30	1,000	*p* < 0.05 (FWE)
Nouwen et al. (2017)	3.0T	FSL	TBSS	MNI	61	1,500	*p* < 0.05 (TFCE)
Xie et al. (2017)	3.0T	SPM, FSL, DKE	VBA	MNI	25	1,000, 2,000	*p* < 0.05 (AlphaSim)
Yoon et al. (2017)	1.5T	FSL	TBSS	MNI	54	1,000	*p* < 0.05 (Cluster-based thresholding)
Ruan et al. (2017)	3.0T	SPM, FSL	VBA	MNI	64	1,000	*p* < 0.05 (corrected for multiple comparisons)
Liang et al. (2019)	3.0T	PANDA	VBA	MNI	25	1,000	*p* < 0.05 (AlphaSim)
Xiong et al. (2019b)	3.0T	FSL	TBSS	MNI	25	1,250, 2,500	*p* < 0.05 (FWE)
Su et al. (2020)	3.0T	FSL	TBSS	MNI	64	1,000	*p* < 0.05 (TFCE)

*ART2, automated registration toolkit 2; DKE, diffusional kurtosis estimator; FA, fractional anisotropy; FWE, family-wise error; FSL, FMRIB software library; MIDAS, multi-modal imaging data analysis system; MNI, Montreal neurological Institute; PANDA, pipeline for analyzing brain diffusion images; SPM, statistical parametric mapping; Tal, talairach; TBSS, tract based spatial statistics; TFCE, threshold-free cluster enhancement; VBA, voxel-based analysis*.

### Meta-Analysis

**Figure 3:**
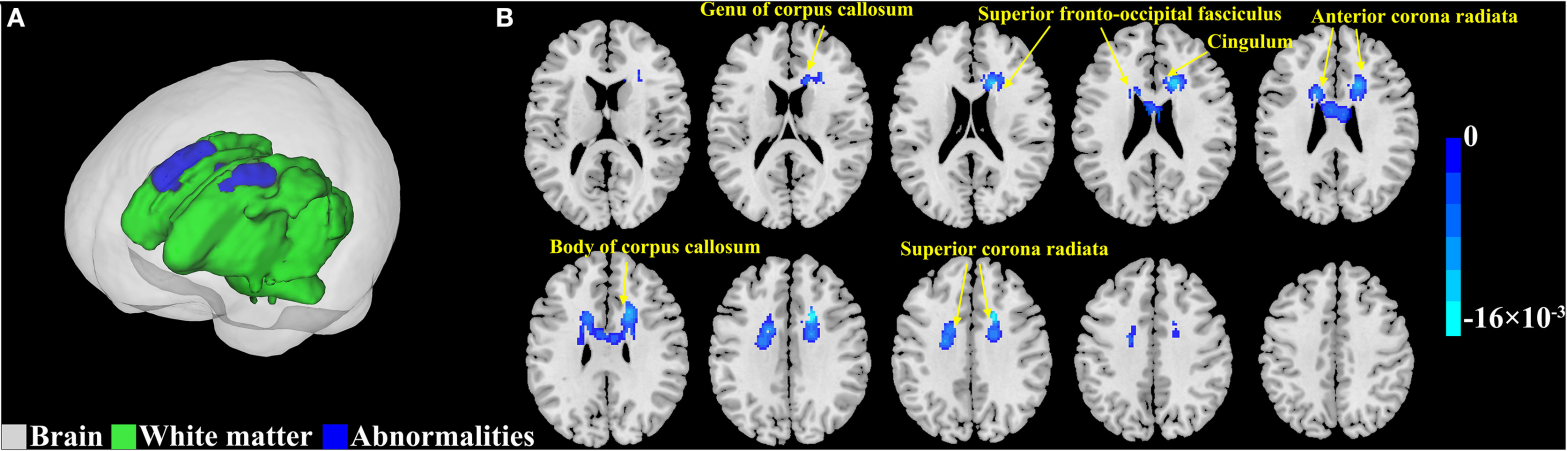
Abnormal white matter estimated by fractional anisotropy (FA) in type 2 diabetes mellitus (T2DM). **(A)** The results from the activation likelihood estimation (ALE) meta-analyses are shown in 3D rendering. The gray region represents the outline of the brain. The green region represents the normal white matter. The blue region represents the abnormalities in the white matter. **(B)** The results from the ALE meta-analyses are overlaid onto a standard template in the Montreal Neurological Institute (MNI) space. The color bar represents the ALE value. Cluster-level inference threshold of *p* < 0.05 [family-wise error (FWE) correction] with 5,000 permutations and *p* < 0.05.

Activation likelihood estimation meta-analysis was performed on the FA values of 381 patients with T2DM from 12 included studies. Compared to controls, patients with T2DM had shown consistent FA reductions (Figure 3; Table 3). The largest cluster (4,432 mm^3^) was located in the body of corpus callosum, and the others included left superior corona radiata (1,944 mm^3^), right superior corona radiata (1,400 mm^3^), right anterior corona radiata (1,336 mm^3^), genu of corpus callosum (448 mm^3^), right cingulum (120 mm^3^), left anterior corona radiata (88 mm^3^), left cingulum (24 mm^3^), left superior fronto-occipital fasciculus (16 mm^3^), and right superior fronto-occipital fasciculus (8 mm^3^).

**Table 3:** Regions that showed consistent fractional anisotropy (FA) reduction in patients with T2DM.

**No**.	**ROI**	**X**	**Y**	**Z**	**Extrema**	**Volume (mm^**3**^)**
1	Genu of corpus callosum	16	20	24	0.01434	448
2	Body of corpus callosum	18	12	32	0.015065	4,432
3	Anterior corona radiata R	18	16	32	0.013598	1,336
4	Anterior corona radiata L	−16	16	30	0.0034261	88
5	Superior corona radiata R	18	12	34	0.017433	1,400
6	Superior corona radiata L	−22	−8	36	0.0094876	1,944
7	Cingulum R	10	20	24	0.0042591	120
8	Cingulum L	−6	−2	32	0.0025344	24
9	Superior fronto-occipital fasciculus R	22	14	22	0.0021478	8
10	Superior fronto-occipital fasciculus L	−20	10	24	0.002947	16

## Discussion

In the present study, a coordinate-based meta-analysis of DTI was conducted to investigate the abnormal white matter in patients with T2DM. Twelve eligible studies with a total of 381 patients with T2DM were analyzed. The consistent abnormal white matters were identified, namely, the commissural fibers genu of corpus callosum and body of corpus callosum, the association fibers cingulum and superior fronto-occipital fasciculus, and the projection fibers anterior corona radiata and superior corona radiata.

Commissural fibers connected the left and right hemispheres, such as corpus callosum, anterior commissure, and fornix. As the largest commissural fiber in the telencephalon, the corpus callosum was an important connective fiber that played a key role in transmitting, integrating, and coordinating information between the left and right hemispheres, whether excitatory or inhibitory (Bloom and Hynd, 2005; Caillé et al., 2005; Roland et al., 2017). Consistent with previous reports (Chen et al., 2013; Yau et al., 2013; Yoon et al., 2017), abnormalities in the corpus callosum were also found in patients with T2DM in this study. Furthermore, based on the discovery of abnormalities in the corpus callosum, we made a more detailed localization and found abnormalities in the genu and body of the corpus callosum. The body of the corpus callosum, which connects the parietal, temporal, and occipital lobes, contains important structures for processing memory, emotions, and execution (Musiek, 1986; Matsukawa et al., 2011; Goldstein et al., 2021). Damage to the body of the corpus callosum would result in dysfunction or abnormal memory, mood, and executive function (Peltier et al., 2012). Impairment in the body of the corpus callosum might play an important role in cognitive impairment in T2DM. Besides, the genu of the corpus callosum contains fibers that connect the left and right prefrontal lobes, the premotor cortex, and the accessory motor cortex (Matsukawa et al., 2011). Abnormalities in the genu and body of the corpus callosum may contribute to sensory, cognitive, mental, or visual impairments in patients with T2DM (Yang et al., 2014). Therefore, these regions may become new targets for the treatment of patients with T2DM who also have cognitive impairment.

Projection fibers are the connecting fibers between the cerebral cortex and the subcortical center. The anterior corona contains projection fibers from the internal capsule to the cerebral cortex (Wakana et al., 2004), which primarily projects to the prefrontal cortex and plays a role in the neural circuitry for emotion regulation (Sanjuan et al., 2013; Goodkind et al., 2015). Consistent with the results of this meta-analysis, several studies on DTI have found that the anterior corona radiata and superior corona radiata were abnormal in patients with T2DM (Xiong et al., 2016, 2019a; Sun et al., 2018). Patients with T2DM are often accompanied by a variety of emotional disorders, such as depression and anxiety. For example, a cross-sectional study found that the prevalence of depression and anxiety in patients with type 2 diabetes in western Saudi Arabia was 33.8 and 38.3%, respectively (Alzahrani et al., 2019). It was reported that the prevalence of anxiety in people with T2DM was significantly higher than in those who were healthy (Tu et al., 2017). At the same time, a meta-analysis showed that almost a quarter of patients with T2DM were depressed (Khaledi et al., 2019). Further studies are needed to explore whether the occurrence of depression and anxiety in patients with T2DM may be related to white matter abnormalities such as the corona radiata.

The abnormal connecting fibers that connect the adjacent gyrus of the ipsilateral cerebral hemisphere were found in T2DM, including the cingulum and superior fronto-occipital fasciculus. Consistent with the previous studies, significant abnormalities in the cingulum of patients with T2DM were found in the study (Liang et al., 2019; Cui et al., 2020). As a core member of the limbic system, the cingulate serves as a bridge connecting the various lobes of the brain and the cingulate gyrus (Dalgleish, 2004; Bubb et al., 2018). The anterior cingulate cortex is related to cognitive control and decision-making, and the posterior cingulate cortex is involved in the adjustment of working memory, visual space, and spatial orientation. In particular, the anterior cingulate cortex plays a key role in error handling (Leech and Sharp, 2014; Bliss et al., 2016; Maldonado et al., 2020). Cingulate impairment in neurological and psychiatric disorders has been widely reported. Based on these studies, we speculate that the abnormal cingulate may be related to cognitive decline in T2DM. In addition, some studies have previously reported the abnormality of the post-default mode network connection in patients with T2DM (Cui et al., 2015; Ishibashi et al., 2018; Liu et al., 2019), and the cingulate as the structural basis of this default network (van den Heuvel et al., 2008). The findings in this study may help to explain these network changes. Interestingly, abnormalities in the fronto-occipital fasciculus were also found in the study, which is consistent with the reduction of FA in bilateral frontal occipital tracts of T2DM reported by Xiong et al. (2019a). The fronto-occipital fasciculus played an important role in visual processing and spatial awareness (Bar et al., 2006; Meola et al., 2015). Therefore, the visual impairment in patients with T2DM might not only be due to retinal atherosclerosis but may also be related to abnormalities of the cingulate and fronto-occipital fasciculus.

This meta-analysis has determined the abnormalities of white matter in patients with T2DM by identifying the consistency of DTI. However, some limitations need to be pointed out. First, the number of included studies was relatively small. Second, few studies have reported the other indicators *via* DTI; therefore, only FA was used to estimate the abnormalities in white matter. Further studies based on MD, AD, and RD should be conducted to comprehensively assess the abnormal white matter in T2DM. Third, on the one hand, the MRI machine, acquisition parameters, and analysis method in each of the studies were different; on the other hand, few studies share the original data because it is too large. It is difficult to estimate the heterogeneous using FA images. Further studies should be done to establish a shared neuroimaging dataset about T2DM, which would make it possible to do the meta-analysis based on the original images.

In summary, the meta-analysis of DTI demonstrated the abnormalities in commissural fibers, association fibers, and projection fibers in T2DM, which might contribute to the neurobehavioral disorders in T2DM. These findings would promote the understanding of the neuropathological mechanisms of T2DM.

## Data Availability Statement

The original contributions presented in the study are included in the article/supplementary material, further inquiries can be directed to the corresponding author/s.

## Author Contributions

SL and JT designed the whole study. LH, TT, MY, and CC searched and selected the studies, analyzed the data, prepared figures, and drafted the article. LH and SL undertook the statistical analysis. LH, QZ, CC, and SL participated in the interpretation of data. LH and QZ wrote the manuscript. SL revised the manuscript. All authors read and approved the final manuscript.

## Conflict of Interest

The authors declare that the research was conducted in the absence of any commercial or financial relationships that could be construed as a potential conflict of interest.

## Publisher's Note

All claims expressed in this article are solely those of the authors and do not necessarily represent those of their affiliated organizations, or those of the publisher, the editors and the reviewers. Any product that may be evaluated in this article, or claim that may be made by its manufacturer, is not guaranteed or endorsed by the publisher.
